# Effect of perioperative blood transfusion on prognosis of patients with gastric cancer: a retrospective analysis of a single center database

**DOI:** 10.1186/s12885-018-4574-4

**Published:** 2018-06-11

**Authors:** Xiaowen Liu, Mingze Ma, Hua Huang, Yanong Wang

**Affiliations:** 1 Department of Gastric Cancer Surgery, Fudan University Shanghai Cancer Center, 270 Dong An Road, Shanghai, 200032 People’s Republic of China; 2 Department of Oncology, Shanghai Medical College, Fudan University, Shanghai, 200032 China

**Keywords:** Gastric cancer, Perioperative blood transfusion, Prognosis

## Abstract

**Background:**

The association between perioperative blood transfusion and the prognosis of patients with gastric cancer is still unclear.

**Methods:**

A total of 1581 patients with gastric cancer who underwent curative gastrectomy from 2000 to 2008 were evaluated. Perioperative blood transfusion was defined as the transfusion of packed red blood cells within seven days before surgery, during surgery, or within the postoperative hospitalization period. The association between perioperative blood transfusion and prognosis was evaluated using univariate and multivariate Cox regression analyses.

**Results:**

Of 1581 patients, 298 patients (19%) received perioperative blood transfusion. Perioperative blood transfusion correlated with older age (*P* < 0.001); larger tumor size (*P* < 0.001); and more advanced stage (*P* < 0.001). Five-year survival rate was 40% in patients who had perioperative blood transfusion and 55% patients who did not have perioperative blood transfusion, and the difference was statistically significant (*P* < 0.001). Multivariate analysis showed that perioperative blood transfusion was defined as independent prognostic factor. Perioperative blood transfusion was associated with worse outcomes in patients with stage III (*P* < 0.001).

**Conclusions:**

Perioperative blood transfusion independently correlated with poorer prognosis in patients with gastric cancer.

## Background

Although the incidence of gastric cancer has declined, it is still the sixth most frequent cancer and the fourth most common cause of cancer death worldwide [1]. In the United States, most patients with gastric cancer are diagnosed at late stage [2]. Anemia is more likely to exist in patients with advanced gastric cancer. Some studies reported that up to 60% of patients presented with perioperative anemia, and most of them undergoing gastrectomy needed red blood cell transfusion [3, 4]. It is well-known that blood transfusions are associated with some adverse outcomes. In particular, some studies showed that blood transfusions were associated with an increased risk of postoperative morbidity [5, 6]. Additionally, some studies have shown that perioperative blood transfusion correlated with poor prognosis of patients with lung cancer, breast cancer, and colorectal cancer [7–9].

Although there have been some studies about the influence of perioperative blood transfusion on prognosis of patients with gastric cancer after undergoing curative gastrectomy, the results still remains controversial [10–13]. Two studies demonstrated that perioperative blood transfusion was associated with worse clinical outcomes for patients with gastric cancer underwent gastrectomy [10, 11]. In contrast, some other studies have not shown worse outcomes [12, 13].

The purpose of this study is to clarify the effect of perioperative blood transfusion on the prognosis of patients with gastric cancer by analyzing large retrospective sample from our institution.

## Methods

### Patients

From 2000 to 2008, 1581 patients with histologically confirmed primary gastric adenocarcinoma underwent curative gastrectomy. Perioperative blood transfusion was defined as the transfusion of packed red blood cells within seven days before surgery, during surgery, or within the postoperative hospitalization period. Postoperative hospitalization is defined as the immediate postoperative period following surgery. Data were retrieved from operative and pathological reports, and follow-up data were obtained by phone, out-patient and clinical database [14]. Written informed consent had been obtained from all the patients, and this study was approved by the Ethical Committee of Fudan University Shanghai Cancer Center. Staging was carried out according to the American Joint Committee on Cancer TNM (Tumor Node Metastasis) Staging Classification for Carcinoma of the Stomach (Seventh Edition, 2010).

### Follow-up

The standard follow-up protocol for patients with gastric cancer was every three months for at least two years, every six months for the next three years, and after five years every 12 months for life [14]. The follow-up items were as follows: physical examination, tumor-marker examination, chest radiography, endoscopic examination, and computed tomographic scan.

### Statistical analysis

The Chi-square test was used to analyze patients’ features and clinicopathological characteristics. The Kaplan-Meier method was used to calculate five-year survival rate, and the long-rank test was used to examine the differences between survival curves. The prognostic factors were included into the multivariate survival analysis using Cox proportional hazards model. The level of significance was *P* < 0.05. Statistical analyses and graphics were carried out using the SPSS 13.0 statistical package (SPSS, Inc., Chicago, IL).

## Results

### Clinicopathological characteristics

There were 1102 males and 479 females (2.3:1) with a mean age of 58 years. According to tumor location, 563 (36%) had tumors located in the upper third; 275 (17%) in the middle third; 702 (44%) in the lower third, and 41 (3%) occupied two-thirds or more of stomach. The distribution of pathological stage was as follows: 403 (26%) patients had stage I, 382 (24%) patients had II, and 796 (50%) patients had III. Patients demographics were listed in Table 1.

**Table 1:** Patient Cohort

	*n* = 1581	100%
Gender		
Male	1102	70
Female	479	30
Age (yr)		
≤ 60	891	56
>60	690	44
Tumor size (cm)		
≤ 5	1136	72
>5	445	28
Tumor location		
Upper third	563	36
Middle third	275	17
Lower third	702	44
Two-third or more	41	3
TNM stage		
Stage I	403	26
Stage II	382	24
Stage III	796	50
Type of Gastrectomy		
Subtotal	1342	85
Total	239	15
Operation time (min)		
< 180	1025	65
≥ 180	556	35
Albumin level at admission (g/dl)		
< 3.5	379	24
≥ 3.5	1202	76
Hemoglobin level at admission (g/dl)		
< 12	575	36
≥ 12	1006	64
Perioperative blood transfusion		
Yes	298	19
No	1283	81

*TNM* Tumor Node Metastasis, *n* number of patients, *min* minute

Clinicopathologic parameters were compared between patients who underwent perioperative blood transfusion and who did not. Results showed that patients with perioperative blood transfusion presented at an older age (*P* < 0.001); larger tumor size (*P* < 0.001); and more advanced stage (*P* < 0.001) (Table 2).

**Table 2:** Comparison of the clinicopathological characteristics of patients with perioperative blood transfusion and without perioperative blood transfusion

Variables	Group with perioperative blood transfusion *n* = 298	Group without perioperative blood transfusion *n* = 1283	*P* value
Gender			0.749
Male	210	892	
Female	88	391	
Age (yr)			< 0.001
≤ 60	119	772	
>60	179	511	
Tumor size (cm)			< 0.001
≤ 5	148	988	
>5	150	295	
Tumor location			< 0.001
Upper third	116	447	
Middle third	68	207	
Lower third	95	607	
Two-third or more	19	22	
TNM stage			< 0.001
Stage I	37	366	
Stage II	84	298	
Stage III	177	619	
Type of Gastrectomy			< 0.001
Subtotal	221	1121	
Total	77	162	
Operation time (min)			0.001
< 180	168	857	
≥ 180	130	426	
Albumin level at admission (g/dl)			0.001
< 3.5	94	285	
≥ 3.5	204	998	
Hemoglobin level at admission (g/dl)			< 0.001
< 12	211	364	
≥ 12	87	919	

*TNM* Tumor Node Metastasis, *n* number of patients; *P* value obtained by chi-squares tests or Fisher’s exact test, *min* minute

### Amount of blood transfusion

Of the 1581 patients, 298 patients (19%) received perioperative blood transfusion. With regard to period and amount of transfusion, 128 (43%) patients received transfusion before operation, 215 (72%) during the operation, and 119 (40%) after the operation. 29 (10%) patients received transfusion only before operation, 105 (35%) only during the operation, and 35 (12%) only after the operation; 134 (45%) patients received less than 4 units, and 164 (55%) patients received more than 4 units.

### Univariate analysis

**Fig. 1:**
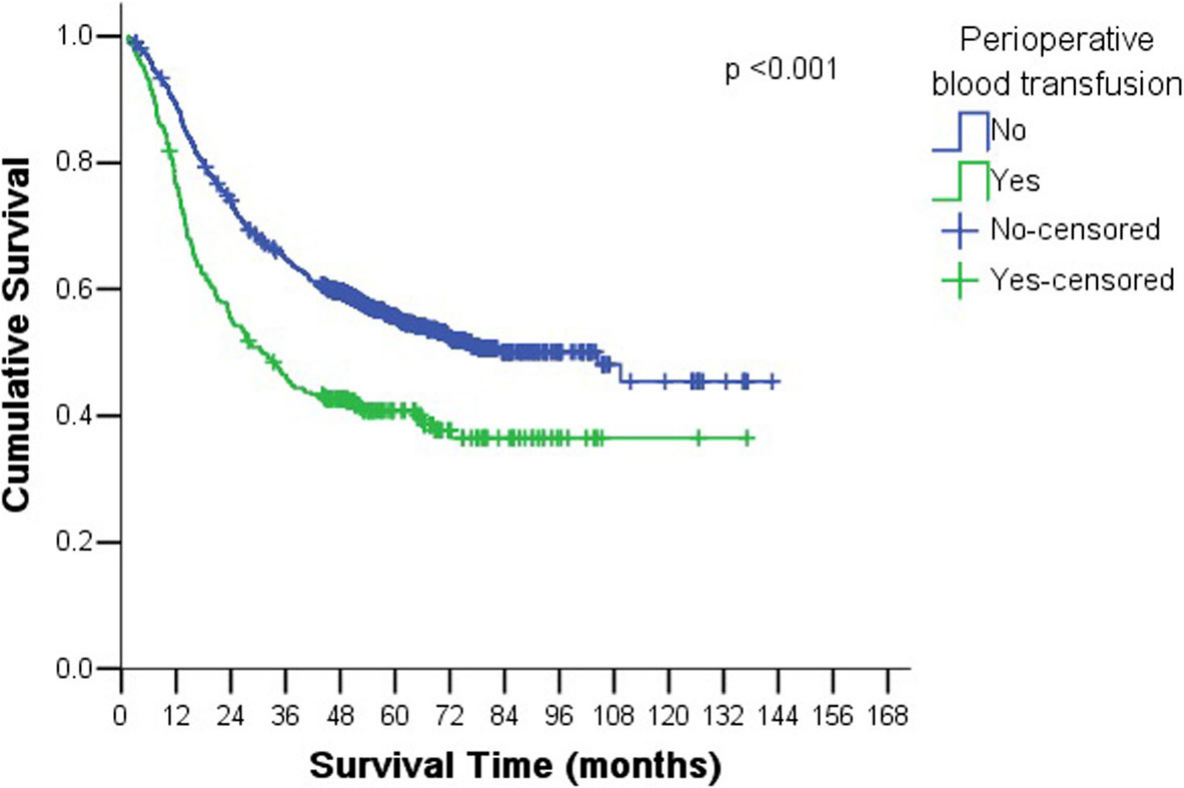
There was significant difference in the survival between group with perioperative blood transfusion and group without perioperative blood transfusion

The median follow-up time was 60.2 months. The over-all five-year survival rate was 53% for all 1581 patients. Five-year survival rate was 40 and 55% in group with perioperative blood transfusion and group without perioperative blood transfusion, respectively, and the difference was statistically significant (*P* < 0.001) (Fig. 1). In addition to perioperative blood transfusion, significant prognostic factors included: age, tumor size, tumor location, TNM stage, type of gastrectomy, operation time, albumin level at admission, and hemoglobin level at admission (Table 3). In patients with perioperative blood transfusion, univariate analysis showed that tumor location and TNM stage significantly affected prognosis, other factors like blood transfusion frequency and blood transfusion amount did not correlate with prognosis (Table 4).

**Table 3:** Univariate analysis of all patients

	n	5-year survival rate (%)	*P* value
Gender			0.759
Male	1102	52	
Female	479	53	
Age (yr)			< 0.001
≤ 60	891	58	
>60	690	45	
Tumor size (cm)			< 0.001
≤ 5	1136	59	
>5	445	36	
Tumor location			< 0.001
Upper third	563	39	
Middle third	275	49	
Lower third	702	65	
Two-third or more	41	32	
TNM stage			< 0.001
Stage I	403	94	
Stage II	382	61	
Stage III	796	27	
Type of Gastrectomy			< 0.001
Subtotal	1342	56	
Total	239	34	
Operation time (min)			< 0.001
< 180	1025	58	
≥ 180	556	43	
Albumin level at admission (g/dl)			0.006
< 3.5	379	47	
≥ 3.5	1202	54	
Hemoglobin level at admission (g/dl)			< 0.001
< 12	575	46	
≥ 12	1006	56	
Perioperative blood transfusion			< 0.001
Yes	298	40	
No	1283	55	

*TNM* Tumor Node Metastasis, *n* number of patients, *P* value obtained by chi-squares tests or Fisher’s exact test, *min* minute

**Table 4:** Univariate analysis of patients with perioperative blood transfusion

	n	5-year survival rate (%)	*P* value
Gender			0.838
Male	210	41	
Female	88	39	
Age (yr)			0.411
≤ 60	119	43	
>60	179	38	
Tumor size (cm)			0.103
≤ 5	148	44	
>5	150	36	
Tumor location			0.035
Upper third	116	35	
Middle third	68	35	
Lower third	95	51	
Two-third or more	19	32	
TNM stage			< 0.001
Stage I	37	89	
Stage II	84	57	
Stage III	177	22	
Type of Gastrectomy			0.060
Subtotal	221	42	
Total	77	34	
Operation time (min)			0.057
< 180	168	45	
≥ 180	130	34	
Albumin level at admission (g/dl)			0.245
< 3.5	94	35	
≥ 3.5	204	42	
Hemoglobin level at admission (g/dl)			0.655
< 12	211	41	
≥ 12	87	38	
Frequency of blood transfusion			0.434
< 2	169	42	
≥ 2	129	37	
Amount of blood transfusion (unit)			0.287
< 4	134	43	
≥ 4	164	38	

*TNM* Tumor Node Metastasis, *n* number of patients, *P* value obtained by chi-squares tests or Fisher’s exact test, *min* minute

### Multivariate analysis

Multivariate survival analysis was performed to determine the independent prognostic factors for patients with gastric cancer. Multivariate analysis showed that age, tumor location, TNM stage, type of gastrectomy, and perioperative blood transfusion were independent prognostic factors (Table 5). In patients with perioperative blood transfusion, multivariate analysis showed that only TNM stage was independent prognostic factor (Table 6).

**Table 5:** Multivariate analysis of patients by Cox model

Variable	Wald	P value	RR	95% CI
Gender	0.419	0.518	1.056	0.895–1.245
Age	7.192	0.007	1.230	1.057–1.431
Tumor location	9.187	0.002	0.879	0.808–0.955
TNM stage	161.018	< 0.001	3.151	2.639–3.762
Type of gastrectomy	12.311	< 0.001	1.403	1.161–1.696
Perioperative blood transfusion	5.385	0.020	0.799	0.661–0.966

*TNM* Tumor Node Metastasis, P value obtained by chi-squares tests or Fisher’s exact test, *RR* relative risk, *CI* confidence interval

**Table 6:** Multivariate analysis of patients with perioperative blood transfusion by Cox model

Variable	Wald	*P* value	RR	95% CI
Gender	0.839	0.360	0.859	0.621–1.189
Age	0.690	0.406	1.138	0.839–1.545
Tumor location	0.942	0.332	0.929	0.801–1.078
TNM stage	59.565	< 0.001	3.268	2.419–4.415

*TNM* Tumor Node Metastasis, P value obtained by chi-squares tests or Fisher’s exact test, *RR* relative risk, *CI* confidence interval

### Comparison of survival according to perioperative blood transfusion at same stage

**Fig. 2:**
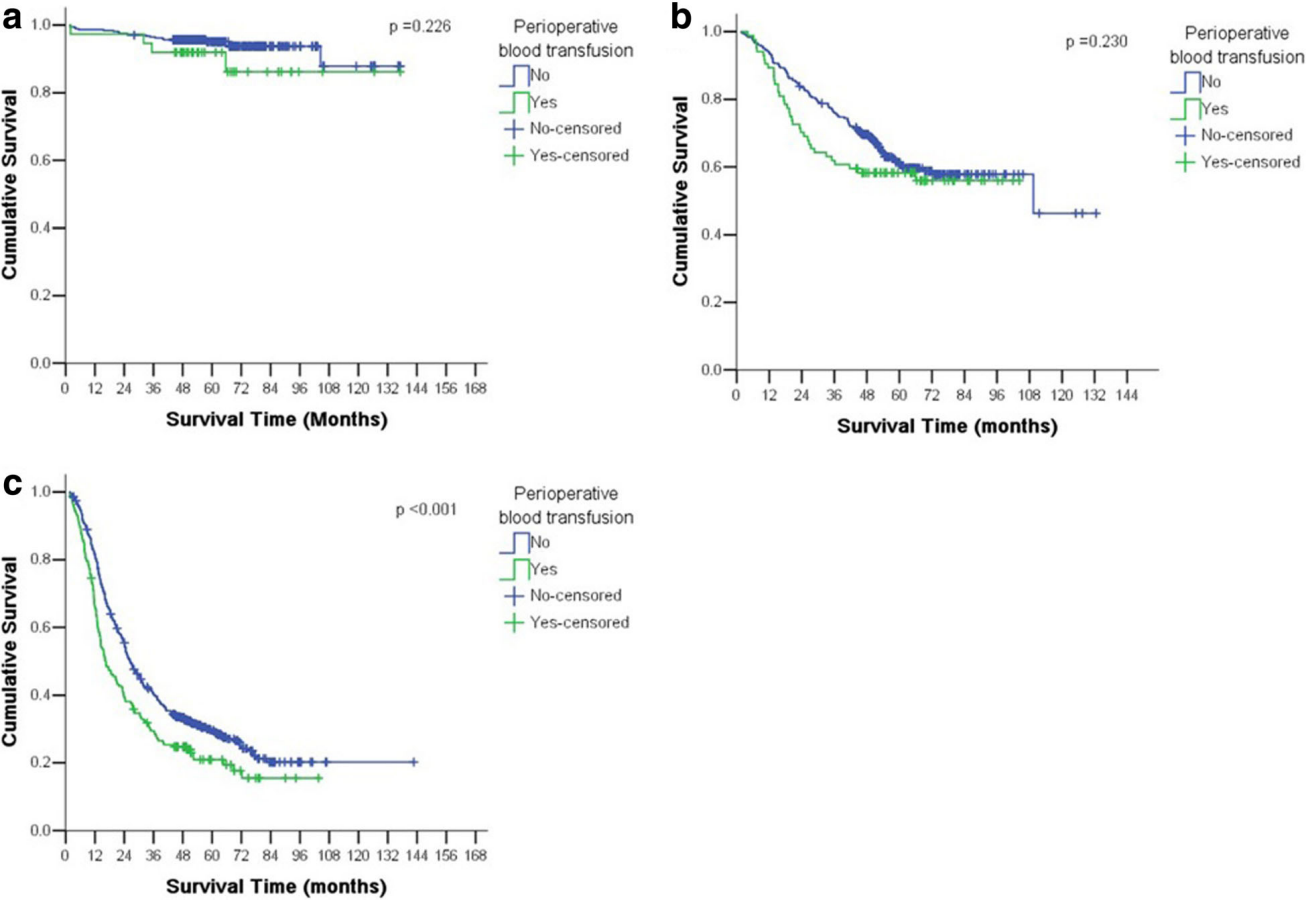
Comparison of survival according to perioperative blood transfusion in same stage. **a** There was no significant difference in patients with stage I. **b** There was no significant difference in patients with stage II. **c** There was significant difference in patients with stage III

Patients with gastric cancer were analyzed by stage (I, II, or III) and whether they underwent perioperative blood transfusion. Patients with gastric cancer were divided into three stages: stage I, stage II, and stage III. Based on perioperative blood transfusion, each stage was divided into group with perioperative blood transfusion and group without perioperative blood transfusion. There was a significant difference of over-all 5-year survival between group with perioperative blood transfusion and group without perioperative blood transfusion according to stage III (*P* < 0.001) (Fig. 2).

## Discussion

The association between overall prognosis and perioperative blood transfusions has been investigated in several solid tumors [15–20]. However, the results have been inconsistent. Two studies have demonstrated that perioperative blood transfusion was associated with worse clinical outcome in patients undergoing gastrectomy, though other studies have not [10, 11]. Stefano Rauseiet al.’s study showed that perioperative blood transfusion did not influence the survival of patients with gastric cancer [12]. Moriguchi et al. reported that there was no relationship between perioperative blood transfusion and survival of patients with gastric cancer [13]. Some reasons should be taken into account of the conflicting results. First, influence of perioperative blood transfusion might be coincidental with other factors, which could result in more blood loss and more transfusions. The present study showed that patients with older age, larger tumor size, and more advanced stage were more likely to receive perioperative blood transfusion, which was consistent with other results [21, 22]. Second, most of the published studies were small-size sample, which had small statistic power to get a positive relationship. Therefore, the present study was carried out in a large-scale sample to avoid the above-mentioned limitations.

In this study, perioperative blood transfusions were associated with a worse prognosis in patients with gastric cancer following gastrectomy. Transfusion was an independent prognostic factor confirmed by Cox regression analysis. In subgroup analysis, the difference in overall 5-year survival was significant for patients with Stage III disease, but not Stage I or II. This finding is consistent with results reported by Xue L et al. [23]. Additionally, we analyzed the relationship between frequency of blood transfusion, amount of blood transfusion, and prognosis. Results showed frequency and amount of blood transfusion did not correlate with the survival, which is consistent with other studies [11, 21, 24]. Therefore, it was possible that blood transfusion itself resulted in poor prognosis rather than frequency and amount of blood transfusion. Despite restrictive usage of blood transfusion is recommended by clinical guidelines, perioperative blood transfusion is still overused in clinical practice.

Although the exact mechanism is not clear, immunosuppression may explain the association between worse overall survival and perioperative blood transfusion. Immunosuppresion can be caused by decreased natural killer cell activity and increased suppressor T lymphocytes activity [25]. Other suppressor factors such as anti-idiotypic antibodies can be produced after receiving blood transfusion [26]. In addition, blood transfusion could promote the proliferation of tumor cells through inducing angiogenesis [27]. This theory was confirmed by Patel et al.’ finding that blood transfusion stimulated proliferation and angiogenesis of endothelial cells [28].

Although the present study is one of the largest retrospective studies in China, there are still some limitations in our study. First, we have not analyzed the effect of blood transfusion on postoperative complications and tumor recurrence. Second, adjuvant radiotherapy and chemotherapy were not included into the analysis. Therefore, it is necessary to carry out prospective, randomized, controlled studies to investigate the prognostic effect of blood transfusion in patients with gastric cancer.

## Conclusions

In conclusion, perioperative blood transfusion independently correlated with poorer prognosis in patients with gastric cancer.
